# Comparison of Human Memory CD8 T Cell Responses to Adenoviral Early and Late Proteins in Peripheral Blood and Lymphoid Tissue

**DOI:** 10.1371/journal.pone.0020068

**Published:** 2011-05-27

**Authors:** Amita Joshi, Biwei Zhao, Cara Romanowski, David Rosen, Phyllis Flomenberg

**Affiliations:** 1 Department of Microbiology and Immunology, Thomas Jefferson University, Philadelphia, Pennsylvania, United States of America; 2 Department of Medicine, Thomas Jefferson University, Philadelphia, Pennsylvania, United States of America; 3 Department of Otolaryngology, Thomas Jefferson University, Philadelphia, Pennsylvania, United States of America; New York University, United States of America

## Abstract

Treatment of invasive adenovirus (Ad) disease in hematopoietic stem cell transplant (SCT) recipients with capsid protein hexon-specific donor T cells is under investigation. We propose that cytotoxic T cells (CTLs) targeted to the late protein hexon may be inefficient *in vivo* because the early Ad protein E3-19K downregulates HLA class I antigens in infected cells. In this study, CD8+ T cells targeted to highly conserved HLA A2-restricted epitopes from the early regulatory protein DNA polymerase (P-977) and late protein hexon (H-892) were compared in peripheral blood (PB) and tonsils of naturally infected adults. In tonsils, epitope-specific pentamers detected a significantly higher frequency of P-977+CD8+ T cells compared to H-892+CD8+ T cells; this trend was reversed in PB. Tonsil epitope-specific CD8+ T cells expressed IFN-γ and IL-2 but not perforin or TNF-α, whereas PB T cells were positive for IFN-γ, TNF-α, and perforin. Tonsil epitope-specific T cells expressed lymphoid homing marker CCR7 and exhibited lower levels of the activation marker CD25 but higher proliferative potential than PB T cells. Finally, in parallel with the kinetics of mRNA expression, P-977-specific CTLs lysed targets as early as 8 hrs post infection. In contrast, H-892-specific CTLs did not kill unless infected fibroblasts were pretreated with IFN-γ to up regulate HLA class I antigens, and cytotoxicity was delayed until 16–24 hours. These data show that, in contrast to hexon CTLs, central memory type DNA polymerase CTLs dominate the lymphoid compartment and kill fibroblasts earlier after infection without requiring exogenous IFN-γ. Thus, use of CTLs targeted to both early and late Ad proteins may improve the efficacy of immunotherapy for life-threatening Ad disease in SCT recipients.

## Introduction

Adenoviruses (Ads) may cause invasive, life-threatening infections in hematopoietic stem cell transplant (SCT) recipients, especially in the pediatric population, such as hepatitis, pneumonitis, colitis, nephritis, and encephalitis [1], [2]. The profound immune suppression following allogeneic bone marrow transplantation, due to ablation of host T cell immunity and administration of immunosuppressive drugs to prevent or treat graft vs. host disease makes patients susceptible to both reactivation of latent Ad infections and primary Ad infections [3]. Group C serotypes (Ad types 1, 2, and 5) common in the general population and the uncommon group B serotypes (Ad types 11 and 35) have most frequently been associated with disease post-SCT, but infections with most serotypes have been described [4], [5]. Although treatment with the toxic antiviral cidofovir may inhibit Ad replication, patients need to develop cell-mediated immunity to recover from invasive Ad disease [6]–[8].

Given the limited therapeutic options, donor lymphocyte infusions have been under investigation as a treatment for Ad disease in allogeneic SCT recipients. As a precedent, infusions of donor lymphocytes or virus-specific T cells have been effective in the treatment of Epstein Barr virus-associated lymphoproliferative disease and cytomegalovirus infections in SCT recipients [9], [10]. Case reports of donor lymphocyte infusions have suggested some benefit against invasive Ad disease [11]–[13]. More recently, Ad-specific T cells, isolated by collecting IFN-γ-secreting T cells after short *in vitro* stimulation with Ad lysate, were infused into 8 pediatric SCT pediatric recipients with Ad infections [14]. Patients were also treated with cidofovir. Four of 5 patients with diarrhea alone or no symptoms resolved their infections, whereas all 3 patients with disseminated Ad disease died. In another study, 11 SCT recipients were treated with donor lymphocytes stimulated *in vitro* with autologous lymphoblastoid cell lines infected with an E1-deleted Ad [15]. Ad-specific T cell responses were detected only in patients (5) with positive Ad cultures. Reductions in Ad viral loads were documented in 3 pediatric patients, and clinical improvement noted in one patient with Ad pneumonia.

A detailed understanding of the T cell response to Ad is required to identify antigen-specific donor T cells that have rapid responses, high proliferation potential, and longevity in order to optimize immunotherapy strategies. Recent advances in T cell biology have shown that two main cell types are involved in memory CD8+ T cell responses – effector memory cells (Tem) (CD62L-/CCR7-) and central memory cells (Tcm) (CD62L+/CCR7+) [16], [17]. Tcm are concentrated in secondary lymphoid tissue and have limited effector functions, but they are highly efficient in self renewal and divide in response to homeostatic cytokines including IL-7 and IL-15. Tem, on the other hand, migrate to peripheral tissues and exhibit pronounced immediate cytolytic (effector) activity but only modest proliferation upon antigenic stimulation. Together, Tem and Tcm contribute to protective immunity depending on the nature and route of infection. Additional levels of complexity in memory CD8+ T cell phenotypes exist between distinct peripheral tissues and in different infectious models [18]. Such functional, anatomic, and phenotypic heterogeneity in the CD8+ T cell memory pool has significant consequences for immunotherapy strategies. Therefore, it is important to determine both the T cell subset(s) and the viral antigen targets able to generate the most efficacious recall responses to Ads.

Additionally, it is crucial to take into consideration the fact that Ads express unique immune evasion proteins that help the virus evade T cell recognition. Notably, the Ad early region 3 E3-19K glycoprotein specifically downregulates cell surface expression of MHC class I molecules, which inhibits recognition of infected cells by CD8+ T cells [19] E3-19K binds to both class I molecules and TAP proteins in the endoplasmic reticulum, which efficiently prevents class I antigen transport to the cell surface [20]. Thus, elimination of infected cells prior to E3-19K mediated downregulation of MHC class I molecules may be more efficient in controlling Ad replication and dissemination. Targeting cells early after infection may also help avoid the effects of other early Ad proteins such as the E3-10.4K/14.5K RID complex (receptor internalization and degradation) and E3-14.7K that confer resistance to apoptosis [21].

We have previously shown that both early regulatory proteins and late structural proteins from Ad can be recognized by human CD8+ CTLs [22], [23]. Additionally, our lab has identified several HLA-A2 restricted epitopes from the early region 2 protein DNA polymerase (e.g P-977) and late capsid protein hexon (e.g. H-892) that are well conserved among different Ad serotypes. Hexon represents the most abundant viral protein and is synthesized late after infection following DNA replication. Rooney and colleagues have also identified other CD8+ T cell epitopes from hexon [24]. Most groups are now focused on expanding hexon-specific T cells *ex vivo* for immunotherapy of Ad disease in SCT recipients [25]–[27].

The goal of this study was to compare the frequency, memory and activation phenotypes, and functional aspects of early protein DNA polymerase- and late protein hexon-specific CD8+ T cells in both peripheral blood and tonsils directly *ex vivo*. CD8+ T cells targeted to HLA-A2 restricted epitopes P-977 from the DNA polymerase and H-892 from hexon were measured *ex vivo* using epitope-specific pentamers. Activation and lymphocyte homing markers and cytokine expression were compared between early and late protein-specific CD8+ T cells in both PB and tonsils. Additionally, P-977 and H-892 epitope specific CTL lines were generated from PBMC by *in vitro* stimulation with Ad5-infected dendritic cells (DC), followed by stimulation with peptide, to compare the kinetics of cytotoxicity.

## Methods

### Study participants

Buffy coat collections were obtained from healthy adult volunteers through the Thomas Jefferson University Hospital Blood Donor Center. Additionally, 1–2 blood specimens (50 ml each) were obtained from SCT recipients with documented invasive Ad infections. Tonsil samples were obtained from patients undergoing tonsillectomies at Thomas Jefferson University Hospital. The research protocols were approved by the Institutional Review Board. All donors were screened by HLA typing or flow cytometry with HLA A2-specific antibody W6/32 to identify HLA A2-positive donors.

### Purification of lymphocytes

Lymphocytes were purified from PBMC by density gradient centrifugation using Ficoll-Hypaque (Sigma Aldrich, St. Louis, MO). For purification of lymphocytes from tonsils, tonsil tissue was cut into small fragments and cells were dispersed by vigorously agitating sample with the flat end of a 5 ml syringe against bottom of culture dish on ice. The fractionated tonsil specimen and cellular suspension were passed through a 100 µm sieve and rinsed well with cold HBSS, until the tissue was clear. Cells were resuspended by repeated pipetting until all cell clumps dissolved and then underlayed with Ficoll-Hypaque. The cell suspension was spun at 900× g for 30 min without brakes, and mononuclear cells at the interphase were harvested. Lymphocytes were aliquoted in media containing 10% DMSO and stored in liquid nitrogen until use.

### Viruses

Group C Ad5 prototype strain was originally obtained from M. Horowitz, Albert Einstein College of Medicine (Bronx, NY). The Ad5 E3-19K deletion mutant dl704 was obtained from W. Wold, St. Louis University (St. Louis, MO) [28]. Ad virions were purified from virus-infected A549 cell lysates by an affinity chromatography method (Clonetech, Mountain View, CA) as per the manufacturer's instructions. The Ad5 virion preparation was titered by ELISA (Cell Biolabs, San Diego, CA).

### Synthetic peptides

The HLA-A2-restricted epitopes H-892 (LLYANSAHAL) and P-977 (VLAWTRAFV) were previously identified from the Ad5 hexon and DNA polymerase protein sequences, respectively. Crude peptides were synthesized by Research Genetics (Huntsville, AL) or Sigma-Genosys (The Woodlands, Texas). Stock solution (10 mM) for each peptide was prepared in 90% DMSO and stored in small aliquots at −80°C.

### IFN-γ ELISPOT assay

This assay was performed as previously described [29].

### Pentamer staining and flow cytometry assays (CFC)

APC-labeled MHC class I HLA A2 Pro5 ^Tm^ Pentamers with H-892 and P-977 peptides were custom synthesized at ProImmune (Oxford, UK). PE-labeled mAbs to human CCR7, CD25, TNF-α, IL-2, Perforin, INF-γ and FITC-labeled human anti-CD8 were purchased from BD Pharmingen (San Diego, CA). For immunostaining, 1–2×10^6^ lymphocytes were stained with a pre-titered amount of pentamer reagent (HLA A2:H-892 or HLA A2:P-977) at room temperature, shielded from light, for 20 min. HLA A2 negative control Pro5 ^Tm^ Pentamers were included to assess nonspecific binding in flow assays. Cells were washed and then stained with PE-labeled CD8, CD25, or CCR7 mAbs on ice for 20 min. Thereafter cells were washed and fixed in 0.4% formaldehyde solution. Cell surface phenotype analysis was done by gating on the CD8+ pentamer+ population.

### Cytokine analysis

CD8+ T cells were isolated from PBMC or tonsils by negative immunomagnetic selection (Miltenyi Biotec, Auburn, CA), and analyzed by intracellular cytokine staining and flow cytometry. Briefly, 1- 2×10^6^ CD8+ T lymphocytes were incubated with or without peptide for 2 h. Cells were treated with brefeldin A (Golgi Stop, BD Pharmingen) for an additional 4 h and then washed, permeabilized, and fixed using the Cytofix/Cytoperm reagent (BD Pharmingen). PE-conjugated anti-human IFN-γ, TNF-α, perforin and IL-2 mAbs were used to measure cytokine secretion. PE-conjugated isotype control cocktail as well as purified blocking antibody cocktail were used to measure background. Samples were collected on a FACSCalibur flow cytometer (BD Pharmingen), and analysis was performed using CellQuest software.

### Statistical analysis

Differences in values between experimental groups were examined for significance with Graph Pad Prism software using the Student *t* test. A probability (*P*) values <0.05 was considered significant.

### Reverse transcriptase PCR (RT PCR)

The kinetics of mRNA expression from the Ad DNA polymerase and hexon ORFs was measured by infecting fibroblasts with wild type (WT) Ad5 (200 pfu/cell) and harvesting cells between 6–24 h post infection. RNA was extracted from infected cells via affinity chromatography (Qiagen, Valencia, CA) and RT PCR was performed using the Quantiscript™ RT (Qiagen). The following primers were used for amplification of DNA polymerase - (forward): 5′ GACTTCACTCTAGAAAGATGATACTCAGAGAGCCCCTC 3′ and (reverse): 5′ GACTTCACGGATCCTAACTGGAGGAGGCGCCGCA 3′ - and hexon - (forward): 5′ GACTTCACTCTAGAAGGTCAGGAAAATGGATGGG 3′ and (reverse): 5′ GACTTCACTCTAGAGATTCCCCAGTCCTTTTCCTCCC 3′ - to produce 1 Kb and 0.3 Kb products, respectively. A control reaction without RT was run for each time point to ensure that the RT PCR products were RNA specific.

### Preparation of Ad-infected dendritic cells

CD14+ cells were isolated from PBMC by positive immunomagnetic selection (Miltenyi Biotec) per the manufacturer's directions. CD14+ cells (1×10^6^/ml) were placed in a 6-well plate in RPMI 1640 supplemented with 5% human AB sera, HEPES, glutamine, and P/S containing GM-CSF (800 U/ml) (Berlex, Wayne, NJ) and IL-4 (1000 U/ml) (BD Pharmingen). Fresh cytokines were added on day 3. Non-adherent cells (immature DC) were harvested on day 5 and infected with Ad (200 pfu/cell) in 200 µl media for 1.5 h or left untreated. DC were re-plated in 6-well plates, and maturation was induced by incubation with 10 ng/ml IL-1β, 1000 U/ml IL-6, 10 ng/ml tumor necrosis factor-α (TNF-α) (BD Pharmingen), and 1 µg/ml prostaglandin E2 (PGE2) (Sigma Aldrich) for 48 h.

### Generation of peptide-specific CTL lines

Bulk Ad-specific T cells were amplified *in vitro* by co-culture of 10×10^6^ lymphocytes with 2×10^6^ Ad5-infected DCs for one week. Then, cells were re-stimulated with peptide (H892 or P977)-loaded, irradiated PBMC on day 7. IL-2 (20 U/ml) was added to each line on day 8, and every 3 to 4 days thereafter. Specificity was confirmed by IFN-γ ELISPOT and the CTL lines were assayed for cytotoxicity around day 14.

### Cytotoxic T cell assay

Cytotoxicity was measured by a calcein release assay as previously described [29]. HLA-A2-positive MRC-5 fibroblasts (targets) were infected for 6, 8, 12, 16 or 24 h with Ad5 (200 pfu/cell). In some cases, fibroblast targets were pre-treated for 24 h with 150 U/ml of human IFN-γ prior to infection. Targets were labeled with 5 µg/ml calcein (Molecular Probes, Eugene, OR) for 30 min at 37°C, washed extensively, and incubated with effectors in microtiter wells for 3 h. Assays were performed in triplicate using a range of effector/target ratios. Calcein release was measured from supernatants on a Victor 2 1420 multilabel counter (Wallace, Gaithersburg, MD).

### Proliferation assay

Proliferation of lymphocytes *in vitro* was measured using CFSE (Molecular Probes) labeling. Briefly, 20×10^6^ purified CD8+ T cells were labeled with 1.5 µM CFSE in PBS for about 8 min. Cells were then incubated for 10 min at 37°C with pre-warmed FBS to allow for efflux of excess CFSE. Cells were washed vigorously three times in PBS containing 1% FBS, and a day 0 sample was obtained to determine initial labeling. For peptide stimulation experiments, 2×10^6^ CFSE-labeled cells were placed in a 24-well plate and cultured in RPMI with 10% FBS with or without P-977 or H-892 peptides for 4 days.

## Results

### Early and late protein-specific CTLs can be detected in peripheral blood and lymphoid tissue

**Figure 1:**
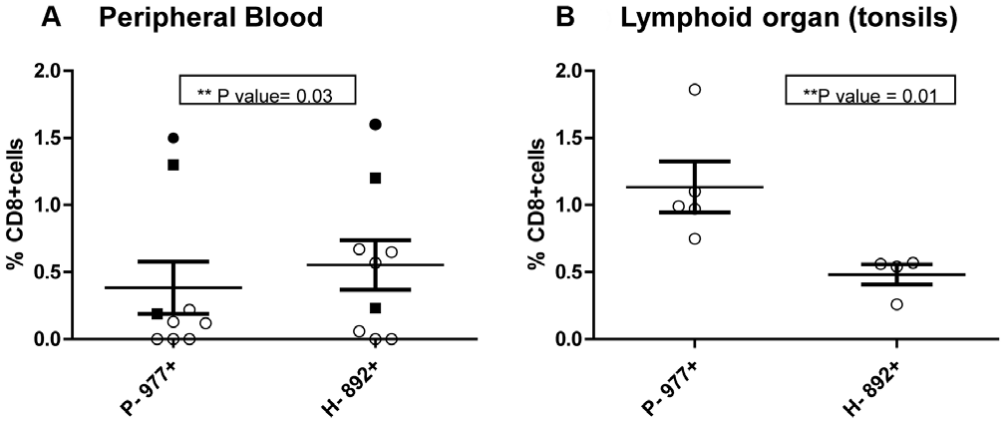
CD8+ T cell responses to DNA polymerase (P-977) and Hexon (H-892) epitopes in peripheral blood and tonsils. Lymphocytes from peripheral blood (PB) or tonsil samples were stained with anti-CD8-FITC mAb and either early protein P-977 or late protein H-892 APC-labeled HLA A2 pentamers and analyzed by flow cytometry. Comparison of the frequencies of P-977+ CD8+ T cells and Hex-892+ CD8+ T cells in all donor samples from PB (**A**) and lymphoid compartments (**B**). Remotely infected adult (○). Recently infected immmunocompromised adult (▪). Recently infected immunocompetent adult (•).

**Figure 2:**
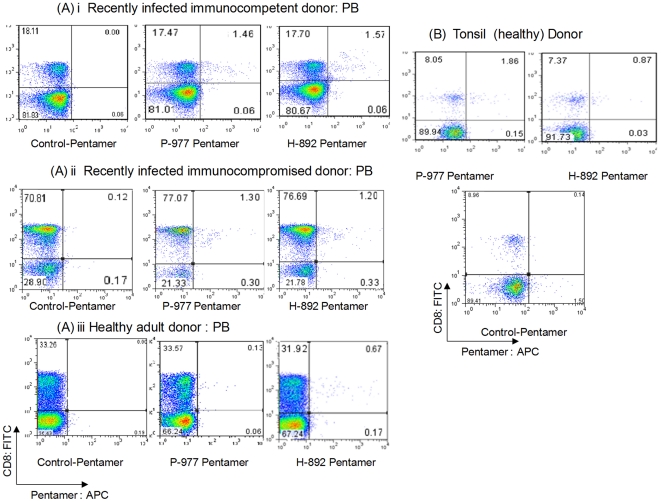
Representative DNA polymerase (P-977) and Hexon (H-892) pentamer staining patterns in peripheral blood and tonsil. Lymphocytes from peripheral blood (PB) or tonsil samples were stained with anti-CD8-FITC mAb and either P-977 or H-892 APC-labeled HLA A2 pentamers and analyzed by flow cytometry. Cells were also stained with HLA A2 negative control pentamers. **A.** PB from a recently infected immunocompetent adult **(i)**, recently infected immunocompromised adult **(ii)**, and remotely infected adult **(iii)**. **B.** Tonsil sample.

Nine HLA A2-positive donors were tested for CD8+ T cell responses to both early and late protein HLA A2-restricted epitopes P-977 and H-892 in PB by *ex vivo* pentamer staining. These epitopes are both highly conserved among different Ad serotypes. PBMC samples were obtained from three different groups of individuals: 1) six healthy adults; 2) one recently infected immunocompetent adult; and 3) two adult SCT recipients who recently recovered from documented invasive Ad infections (2 and 12 months post-infection). All donors had positive T cell responses to Ad lysate by IFN-γ ELISPOT assay (data not shown). Overall, as shown in **Figure 1A**, P-977+CD8+ T cells were detected at lower frequencies than H-892+CD8+ T cells in PB (mean 0.38% vs 0.57%, p = 0.03). Recently infected donors (immunocompromised and immunocompetent) exhibited high, comparable frequencies of P-977- and H-892-specific CD8+ T cells (**Figures 1A**, **2A**). In contrast, the frequencies of P-977+CD8+ T cells were relatively lower than H-892+CD8+ T cells in PB samples from healthy adults. Two of 6 healthy donors did not have detectable H-892 or P-977 responses in PB, while one donor had a low H-892 response but no P-977 response using this method.

Tonsils from 5 of 5 HLA A2+ donors tested exhibited positive responses to one or both epitopes by *ex vivo* pentamer staining (**Figures 1B**, **2B**). Notably, in contrast to PB responses, P-977+CD8+ T cells were detected at significantly higher frequencies than H892+CD8+ T cells in tonsils (mean 1.1% vs. 0.48%, p = 0.01). One tonsil sample exhibited a high frequency of P-977+CD8+ T cells (1.1%) but did not have a detectable H-892 response.

### Early and late CTLs from peripheral blood and tonsils have distinct phenotypes

**Figure 3:**
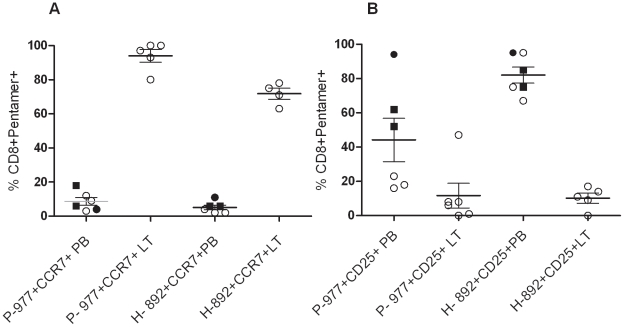
Expression of homing and activation markers in epitope-specific CD8+ T cells from peripheral blood and tonsils. Early P-977 pentamer+ and late H-892 pentamer+ CD8+ T cells from peripheral blood (PB) or lymphoid tissue (LT) samples were stained with either T cell activation marker CD25 or lymph node homing receptor CCR7 PE-labeled mAbs. **A.** Comparison of the proportion of P-977+CD8+ T cells and H-892+CD8+ T cells expressing CCR7 in PB and.LT samples. **B.** Comparison of the proportion of P-977+CD8+ T cells and H-892+ CD8+ T cells expressing CD25 in PB and LT samples. Recently infected immunocompromised adult (▪). Recently infected immunocompetent adult (•). Remotely infected healthy donor (○).

Cell surface expression of the lymph node homing receptor CCR7 and the activation receptor CD25 (IL-2R) was analyzed in PB and tonsil mononuclear cell samples. CD8+ T cells from both early protein P-977 pentamer+ and late protein H-892 pentamer+ populations had negligible cell surface expression of CCR7 in all PB samples (**Figure 3A**). In contrast, CCR7 was highly expressed in tonsil samples by both early and late protein pentamer+ CD8+ T cells. The signature of the T cell activation receptor CD25 was more diversified. PB samples from remotely infected adults showed a distinction between early and late protein epitope-specific T cell populations (**Figure 3B**). P-977+CD8+ T cells from remotely infected adult PB showed low expression of CD25, whereas most H-892+CD8+ T cells were positive for CD25. In comparison, the vast majority of both early and late protein epitope+ CD8+ T cells from recently infected donor PB (both immunocompetent and immunocompromised) exhibited cell surface expression of CD25. Tonsil samples had negligible expression of CD25 from both early and late protein epitope-specific CD8+ T cells consistent with a central memory phenotype, except for 1 tonsil that exhibited moderate expression of CD25 from P-977+CD8+ T cells only.

### Functional profile of peripheral blood and tonsil P-977- and H-892-specific CD8+ T cells

**Figure 4:**
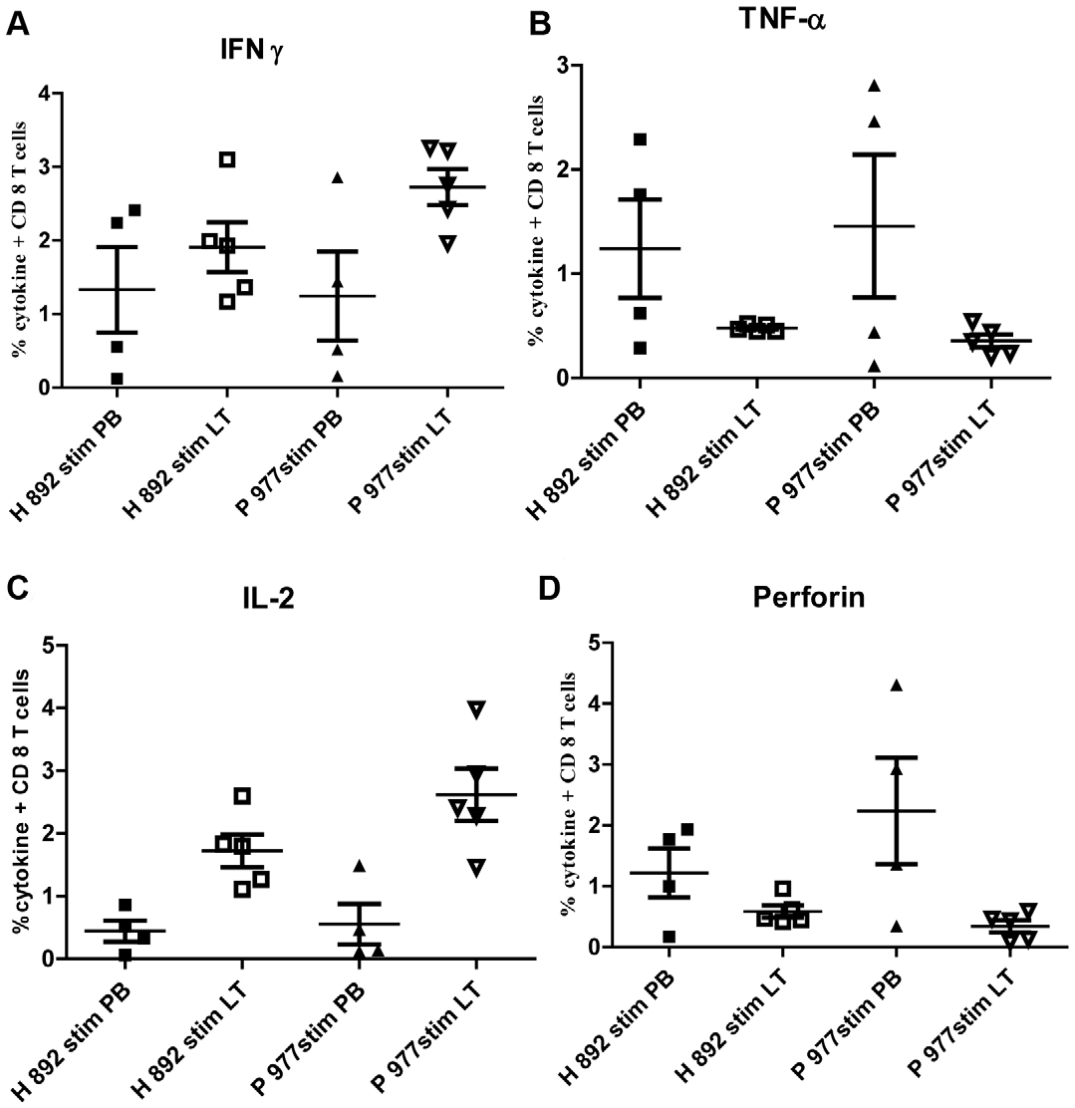
Functional analysis of DNA polymerase and hexon epitope-specific CD8+ T cells from peripheral blood and tonsils. Purified CD8+ T cells from peripheral blood (PB) or lymphoid tissue (LT) samples were stimulated (stim) with either P-977 or H-892 peptides for 6 h. Samples were assayed for intracellular cytokine expression of IFN-γ **(A)**, TNF-α **(B)**, IL-2 **(C)**, and perforin **(D)**. Recently infected immunocompromised adult, H- 892 (▪) or P-977 (•). Remotely infected adult, Hex 892 (□) or P-977 (○).

Cytokine secretion and proliferative potential of early and late protein epitope-specific CD8+ T cells from lymphoid tissue and PB were compared. Within each compartment, early P-977- and late H-892-specific CD8+ T cells had similar cytokine secretion profiles by intracellular cytokine flow analysis. IFN-γ, TNF-α, and perforin were expressed by PB CD8+ T cells stimulated 6 h with either the P-977 or H-892 peptide, but there was relatively little IL-2 secretion **(** **Figure 4**, **Figures S1**, **S2**, **S3**). The cytokine release pattern in tonsils was distinct from that of PB samples. Lymphoid CD8+ T cells had significant release of both IL-2 (which was negligible in PB CTLs) and IFN-γ but expressed little or no perforin and TNF-α when stimulated *in vitro* with either peptide).

**Figure 5:**
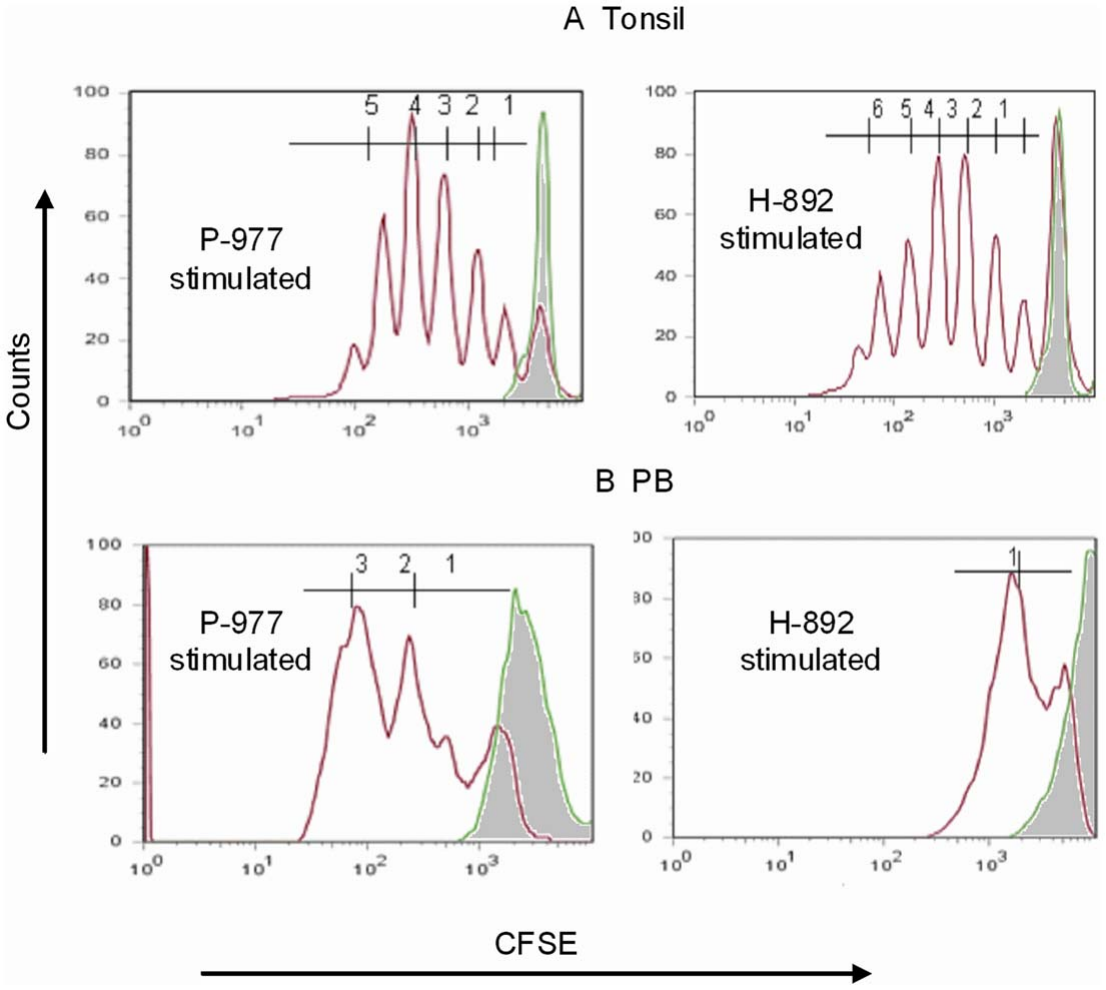
Functional analysis of DNA polymerase and hexon epitope specific CD8 T cells using proliferation assay. CFSE-labeled CD8+ T cells from tonsils **(A)** or PB **(B)** were stimulated with relevant peptides (P-977 or H-892 or media) for 4 days. Flourescence intensity of day 0 (shaded area) and day 4 samples was compared.

The proliferative potential of lymphoid and PB epitope-specific CD8+ T cells was also compared by CFSE staining following *in vitro* antigen exposure for 4 days from single donors (**Figure 5**). Lymphoid CD8+ T cells exhibited a higher proliferative potential than PB CD8+ T cells when stimulated with either P-977 or H-892 peptides. Additionally, within both compartments, early P-977-specific CD8+ T cells had a higher proliferative potential than late H-892 peptide-specific T cells. Thus, early protein P-977 CD8+ T cells from the lymphoid compartment had the highest proliferation potential of all.

### P-977-specific and H-892-specific CTLs have distinct cytotoxicity profiles

**Figure 6:**
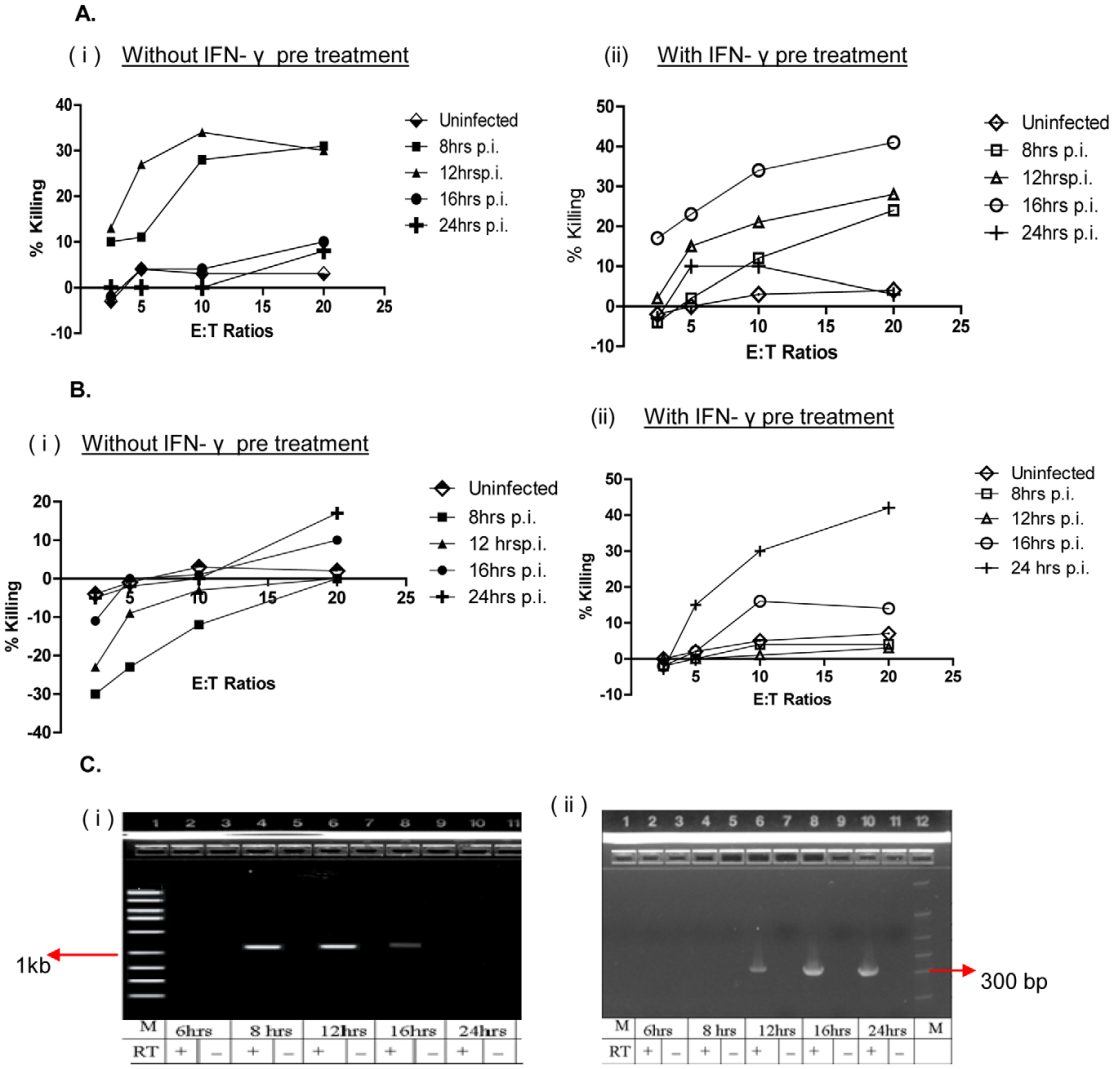
Kinetics of cytotoxicity of DNA polymerase (P-977) and hexon (H-892) specific CTLs. P-977- and H-892-specific CTL lines were tested for cytotoxicity against fibroblast targets infected with wildtype Ad5 using a 3 h calcein release assay. Fibroblasts were infected with Ad5 for 8 to 24 h prior to assay; uninfected fibroblasts were used as a negative control. **A.** Cytotoxicity of P-977-specific CTLs against fibroblast targets without IFN-γ pretreatment **(i)** or pretreated with IFN-γ **(ii)**. **B.** Cytotoxicity of H-892-specific CTLs against fibroblast targets **(i)** without IFN-γ pretreatment or **(ii)** pretreated with IFN-γ. **C.** Expression of DNA polymerase and hexon mRNA was measured in fibroblasts infected with WT Ad5 for 6–24 h. mRNA was amplified by 2 step RT-PCR using primers for **(i)** DNA polymerase and **(ii)** hexon. A control reaction in which cDNA product was amplified in the absence of reverse transcriptase (RT) was included for each time point. M, 1Kb ladder **(i)**; PCR marker ladder (Promega, WI) **(ii)**.

PB lymphocytes from one of the healthy donors were stimulated *in vitro* with Ad-infected DCs for 1 week, followed by stimulation with individual peptides, to generate early protein P-977 and late protein H-892 peptide-specific CTLs (as described in methods). These early P-977 or late H -892 CTLs were incubated with fibroblast targets infected with either WT Ad5 or the Ad5 E3-19K deletion mutant dl704. Targets were infected for 6, 8, 12, 16, or 24 h, and cytotoxicity was measured by calcein release assay (as described). Early P-977 CTLs lysed WT Ad5-infected targets as soon as 8 h post infection (**Figure 6A** **(i)**); cytotoxicity peaked at 12 h and was absent at later time points. In the absence of E3-19K expression, early P-977 CTLs were able to lyse Ad dl704-infected cells efficiently up to 16 h post infection (**Figure S4**). In contrast, late H-892 CTLs did not efficiently kill WT Ad5-infected targets (**Figure 6B** **(i)**). Our lab has previously shown that IFN-γ can help overcome the E3-19K-mediated inhibition of class I cell surface expression [30]. Therefore, fibroblast targets were treated with IFN-γ prior to infection, and the cytotoxicity assays were repeated. As shown in **Figure 6A** **(ii)**, the early P-977 CTLs lysed WT Ad5-infected targets pre-treated with IFN-γ 8 h post infection, and cytotoxicity peaked at 16 h, similar to the kinetics using dl704-infected fibroblasts. Moreover, as shown in **Figure 6B** **(ii)**, late H-892 CTLs efficiently killed WT Ad5-infected targets pre-treated with IFN-γ, but cytotoxicity did not occur until 16–24 h post infection. Similarly, the late H-892-specific CTLs killed Ad dl704-infected fibroblasts without IFN-γ pre-treatment at 16–24 h (**Figure S4**). These data confirm the role of E3-19K in inhibiting CTL recognition of hexon but not DNA polymerase.

Finally, the kinetics of DNA polymerase and hexon expression was studied by RT-PCR analysis of Ad5-infected fibroblasts. Robust expression of DNA polymerase mRNA was observed as early as 8 h post infection and started to decline by 16 h (**Figure 6C**). In contrast, hexon mRNA could not be detected until 12 h following infection and expression peaked at 16–24 h post infection. The kinetics of mRNA expression paralleled the above cytotoxicity data in that early protein P-977 CTLs started killing infected cells at 8 h, whereas late protein H-892 CTL cytotoxicity was delayed until 16 h post infection.

## Discussion

A detailed understanding of Ad-specific T cell responses is needed in order to optimize immunotherapy strategies for SCT recipients infected with Ads, pathogens which may be associated with significant morbidity and mortality [31], [32]. Although most healthy adults exhibit memory T cell responses to Ads, little is known about the kinetics or memory compartmentalization of Ad-specific T cells. This is the first study to identify the phenotype and functional status of Ad-specific CD8+ T cells in tonsils and to compare the responses of secondary lymphoid and PB compartments following natural Ad infection. Additionally, this study compares the characteristics of CTLs targeted to highly conserved epitopes from the early regulatory protein DNA polymerase and late capsid protein hexon in both PB and lymphoid compartments. This study documents that DNA polymerase-specific CTLs kill fibroblasts earlier after infection than hexon-specific CTLs, prior to Ad E3-19K-mediated down regulation of HLA class I expression. Additionally, central memory type CTLs targeted to DNA polymerase dominate the lymphoid compartment and have a higher proliferative potential compared to hexon-specific CTLs.

CD8+ T cell responses to HLA A2-restricted epitopes from DNA polymerase (P-977) and hexon (H-892) were detected in both PB and tonsil samples using epitope-specific pentamers. Recently infected subjects exhibited high, comparable frequencies of P-977+CD8+ and H-892+CD8+ T cells in PB. Although, as expected, remotely infected donors exhibited significantly lower responses to Ad-specific epitopes in PB, H-892+CD8+ T cells were detected at significantly higher levels than P-977+CD8+ T cells. In contrast, there were significantly higher frequencies of P-977+CD8+ T cells compared to H-892+CD8+ T cells in tonsil samples. Thus, early protein DNA polymerase-specific CD8+ T cells dominate the lymphoid compartment, while late protein hexon-specific CD8+ T cells are predominant in PB of remotely infected adults.

Phenotypic analysis of Ad-specific CTLs from PB and tonsils revealed significant differences between compartments, as well as between epitopes. Several reports have described two subsets of memory CD8+ T cells based upon expression of lymph node homing receptors: CD62L+CCR7+CD8+ central memory T cells -Tcm and CD62L-CCR7-CD8+ effector memory T cells -Tem [17], [33]. In PB, both P-977+CD8+ and H-892+CD8+ T cells from recently infected subjects exhibited high level expression of the T cell activation receptor CD25 and were negative for the lymphoid homing marker CCR7. In tonsils, on the other hand, both P-977+CD8+ and H-892+CD8+ T cells exhibited negligible expression of CD25 and high expression of CCR7. Thus, P-977-specific CD25-CCR7+ CD8+ Tcm-like cells predominate in the lymphoid organs while H-892-specific CD25+CCR7-CD8+ Tem-like cells are found mostly in PB. Interestingly, there was a mixed phenotype of Ad-specific CD8+ T cells in PB from remotely infected adults. Although Ad-specific T cells from PB in remotely infected subjects were all CCR7 negative and most P-977+CD8+ T cells did not express CD25, the majority of the higher frequency H-892+CD8+ T cells were CD25+, similar to the phenotype in recently infected adults.

These data suggest that over time following infection, DNA polymerase-specific CTLs preferentially home to secondary lymphoid tissue, while hexon-specific CTLs remain in PB. This is consistent with the fact that DNA polymerase is a regulatory protein that is expressed transiently in active infection, as reflected by the cytotoxicity and RT-PCR kinetics data presented, and thus there is little if any residual antigen in vivo. In contrast, hexon is the major capsid protein in the virion and is over-expressed in Ad-infected cells. Therefore, hexon-specific CTLs likely remain in an activated state in PB due to persistent antigen exposure. Studies have documented the persistence of viral antigens from other lytic viruses such as influenza and vesicular stomatitis virus [34], [35]. More recently, respiratory DCs that migrate to draining lymph nodes were identified as the major source of persistent antigen following acute influenza virus infection [36]. The presence of activated hexon-specific CTLs in PB in remotely infected adults may also reflect persistent low level Ad replication and/or boosting of hexon-specific T cells by re-exposure to exogenous Ads.

The central memory type CTLs (Tcm) are considered the “true” memory subset of CD8+ T cells, and several reports have documented that viral replication *in vivo* is more effectively controlled by CD8+ Tcm [37], [38]. Wherry *et al* also showed that CD8+ Tcm are able to persist long term *in vivo* by self renewal, are able to secrete IFN-γ, and rapidly expand upon re-encounter with pathogen [17]. CD8+ Tcm were also shown to elicit greater protection against experimental tumors after adoptive transfer followed by vaccination [39]. Analysis of cytokine expression and proliferation demonstrated functional differences in Ad-specific T cells primarily based upon the compartment, not the epitope. In PB, most CD8+ T cells targeted to either P-977 or H-892 secreted IFN-γ, TNF-α, and perforin but not IL-2, consistent with a Tem phenotype. These data are consistent with a recent study in which Ad E1-deleted vector-stimulated CD8+ T cells were found to secrete both IFN-γ and perforin; however, TNF-α secretion was not detected [40]. Tonsil T cells targeted to either P-977 or H-892 secreted both IL-2 and IFN-γ but exhibited little or no expression of perforin and TNF-α consistent with a CD8+ Tcm phenotype. Additionally, Tcm-type tonsil CTLs showed higher proliferative potential when stimulated with cognate antigen in vitro than Tem-type PB derived CTLs. In both compartments, however, P-977-specific CTLs exhibited a higher proliferative ability than H-892-specific CTLs.

The fact that lymphoid derived Tcm and DNA polymerase-specific CTLs have a proliferative advantage over hexon-specific CTLs has implications for protective immunity, as more rapid proliferation will eventually lead to greater expansion of this subset *in vivo*. Additionally, only Tcm-type tonsil CTLs produced IL-2, consistent with their greater potential for *in vivo* expansion. Ad-specific Tcm may also be exposed to more efficient antigen presentation *in vivo* as these CTLs home to primary and secondary lymphoid organs. Infusion of large number of Tem-type cells may provide immediate protection but fail to reconstitute long term memory [41]. Thus, for immunotherapy applications, the superior ability of Tcm to reconstitute the memory T cell pool may be highly relevant. Based on other studies, it may also be important to infuse both CD4+ and CD8+ Ad-specific T cells in order to help amplify and maintain CD8+ T cell responses [9], [42],

Notably, P-977 CTLs lysed fibroblasts *in vitro* significantly earlier following infection compared to H-892 CTLs and evaded E3-19K-mediated inhibition of cytotoxicity. In parallel with the kinetics of mRNA expression, P-977 CTLs killed fibroblasts as early as 8 h post infection. In contrast, H-892 CTLs were unable to kill infected fibroblasts unless targets were treated with IFN-γ to up regulate HLA class I antigens prior to infection, and cytotoxicity was delayed until 16–24 hours. This result confirms our prior study in which bulk Ad-specific CTLs did not kill infected fibroblast targets without IFN-γ pre-treatment as a result of E3-19K-mediated down regulation of class I antigens [30]. Thus, CTLs targeted to DNA polymerase may be able to more rapidly and efficiently eliminate Ad-infected cells *in vivo* compared to hexon-specific CTLs. Targeting Ad-infected cells prior to viral DNA replication will also prevent new virus production.

We acknowledge that the small sample size is a major limitation of this study. Unfortunately, acute Ad infections are infrequently identified in adults and the number of adult SCT patients who develop and recover from invasive Ad infections is very limited. It took several years to identify the 3 HLA-A2 positive recently infected donors used in this study. Additionally, we had access to a limited number of PBMC from each donor, especially the recently infected patients, so analyses had to be carefully selected. Although there were statistically significant differences in the precursor frequencies of H-892- and P-977-specific CD8+ T cells in PB and tonsil samples, the differences in cytokine expression and proliferation need to be confirmed in a larger study. Also, these analyses were performed using a single epitope for each protein, which may not necessarily reflect the total CTL response to each protein. Additionally, the intracellular cytokine analysis was done with a single stain at a time, so this study did not characterize the expression of multiple cytokines from individual cells. Finally, although it would be ideal to evaluate the protective potential of these CTLs after adoptive transfer in an animal model, a suitable animal model that mimics human Ad infection is not available, and animal Ads, such as mouse Ads, have significantly different properties [43].

In summary, these data show that in contrast to hexon-specific CTLs, central memory type CTLs targeted to DNA polymerase dominate the lymphoid compartment and have a higher proliferative potential. Additionally, DNA polymerase-specific CTLs kill fibroblasts earlier after infection compared to hexon-specific CTLs, prior to both E3-19K-mediated down regulation of HLA class I expression and Ad replication. In contrast, hexon CTLs cannot recognize Ad-infected cells unless exposed to exogenous IFN-γ to maintain HLA class I expression. Thus, use of CTLs targeted to both early protein DNA polymerase and late protein hexon may improve the efficacy of immunotherapy for life-threatening Ad disease in SCT recipients.

## Supporting Information

Figure S1 **Intracellular cytokine staining of purified CD8+ T cells stimulated with H-892, P-977, or media alone from a representative healthy donor peripheral blood sample.**
(TIF)

Figure S2 **Intracellular cytokine staining of purified CD8+ T cells stimulated with H-892, P-977, or media alone from a recently infected donor peripheral blood sample.**
(TIF)

Figure S3 **Intracellular cytokine staining of purified CD8+ T cells stimulated with H-892, P-977, or media alone from a tonsil sample.**
(TIF)

Figure S4 **Kinetics of cytotoxicity of P-977- and H-892-specific CTL lines against fibroblasts infected with the Ad5 E3-19K deletion mutant dl704.**
(TIF)
