# Supplementary figures and images for: Comparison of Human Memory CD8 T Cell Responses to Adenoviral Early and Late Proteins in Peripheral Blood and Lymphoid Tissue
Source: PLoS One. 2011 May 27;6(5):e20068. doi: 10.1371/journal.pone.0020068 (PMC3103520; doi:10.1371/journal.pone.0020068)

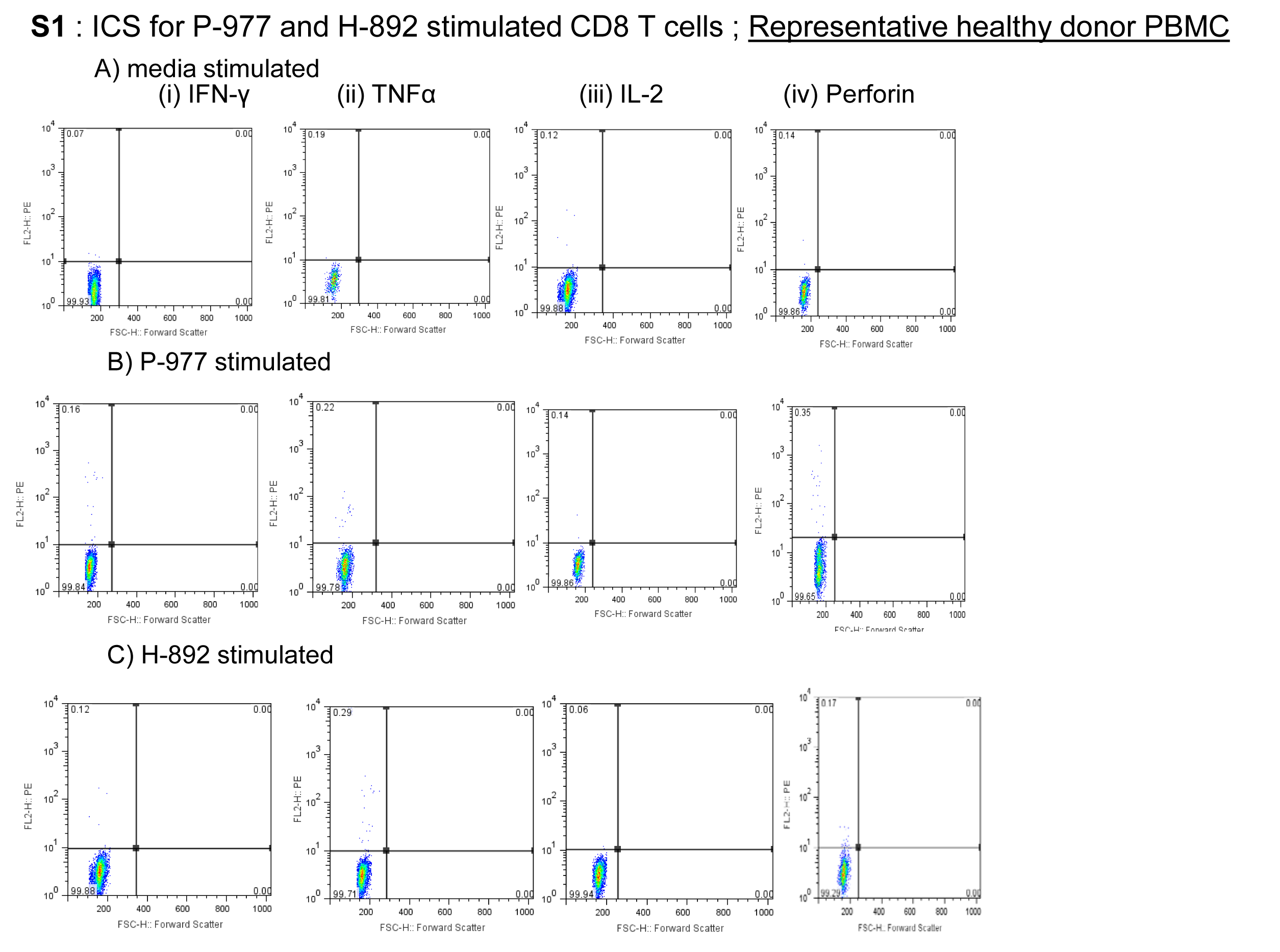

Supplement: Figure S1 — Intracellular cytokine staining of purified CD8+ T cells stimulated with H-892, P-977, or media alone from a representative healthy donor peripheral blood sample. (TIF) [file pone.0020068.s001.tif]

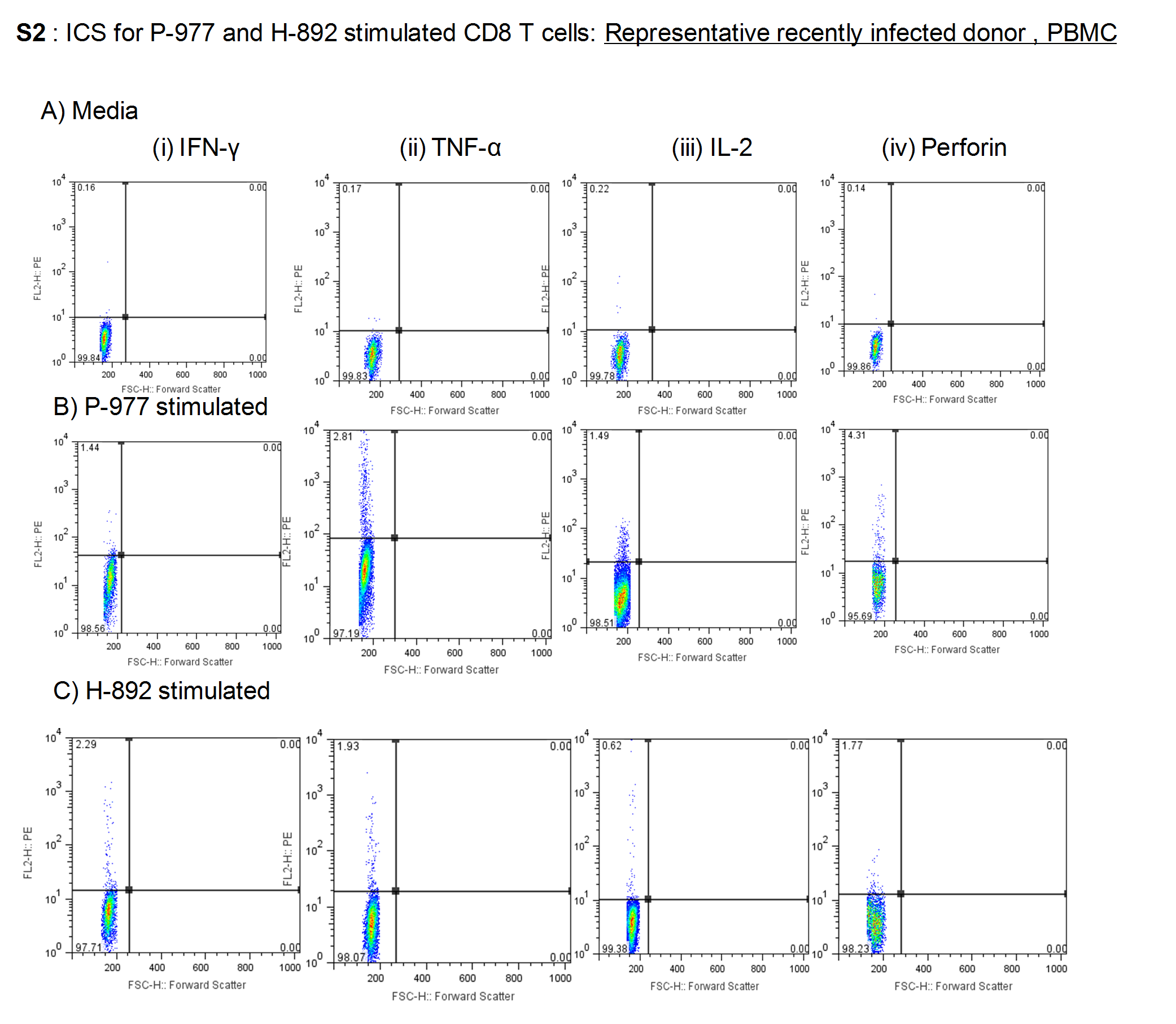

Supplement: Figure S2 — Intracellular cytokine staining of purified CD8+ T cells stimulated with H-892, P-977, or media alone from a recently infected donor peripheral blood sample. (TIF) [file pone.0020068.s002.tif]

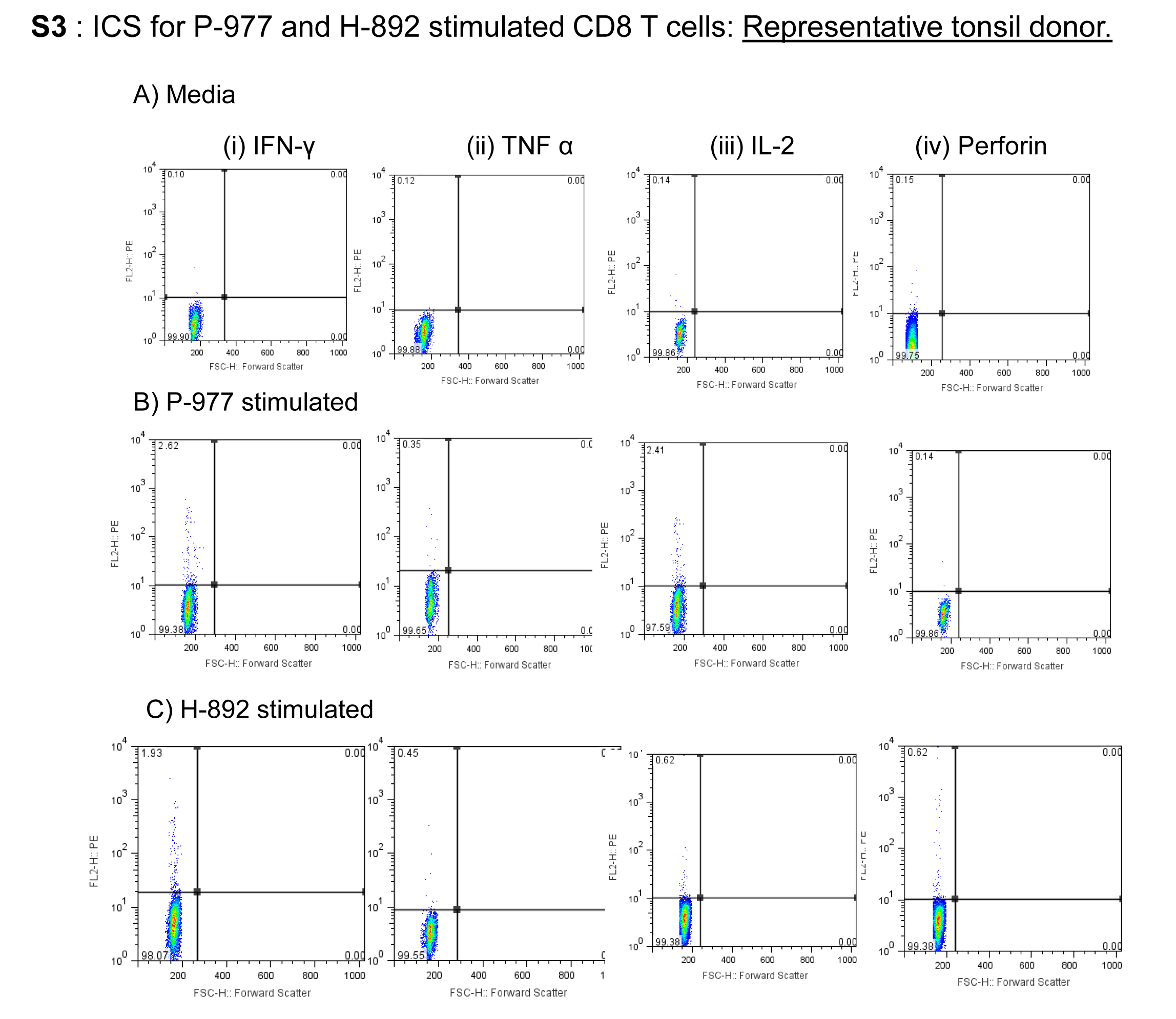

Supplement: Figure S3 — Intracellular cytokine staining of purified CD8+ T cells stimulated with H-892, P-977, or media alone from a tonsil sample. (TIF) [file pone.0020068.s003.tif]

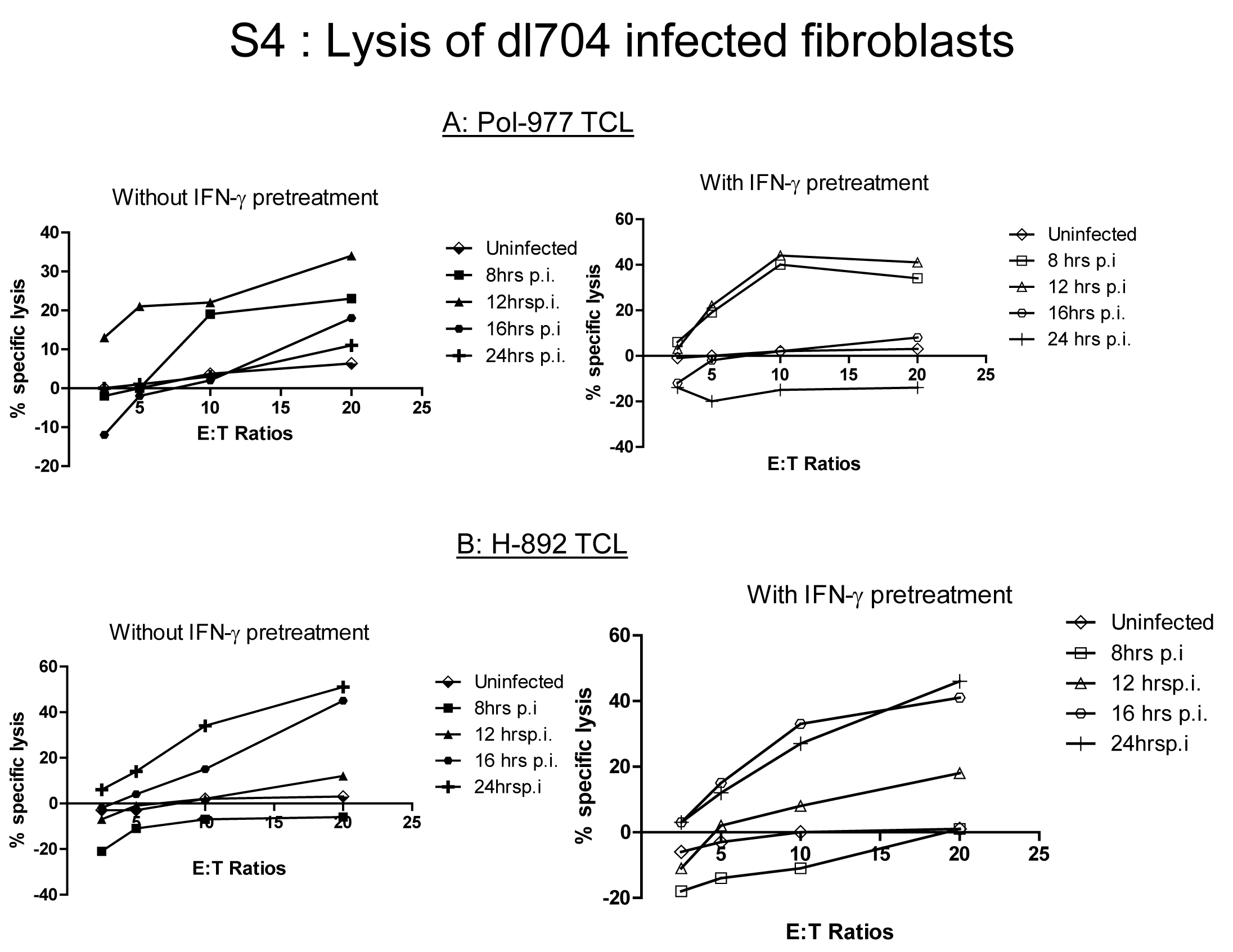

Supplement: Figure S4 — Kinetics of cytotoxicity of P-977- and H-892-specific CTL lines against fibroblasts infected with the Ad5 E3-19K deletion mutant dl704. (TIF) [file pone.0020068.s004.tif]
